# A Role for the Microbiota‐Gut‐Brain Axis in Avoidant/Restrictive Food Intake Disorder: A New Conceptual Model

**DOI:** 10.1002/eat.24326

**Published:** 2024-11-14

**Authors:** Elizabeth Schneider, Ricarda Schmidt, John F. Cryan, Anja Hilbert

**Affiliations:** ^1^ APC Microbiome Ireland, University College Cork Cork Ireland; ^2^ Department of Psychosomatic Medicine and Psychotherapy, Research Unit Behavioral Medicine, Integrated Research and Treatment Center AdiposityDiseases University of Leipzig Medical Center Leipzig Germany; ^3^ German Center for Child and Adolescent Health (DZKJ), partner Site Leipzig/Dresden Leipzig Germany; ^4^ Department of Anatomy and Neuroscience University College Cork Cork Ireland

**Keywords:** avoidant/restrictive food intake disorder, mechanisms, microbiota‐gut‐brain axis, model

## Abstract

**Objective:**

Avoidant/restrictive food intake disorder (ARFID) is an eating disorder characterized by a severely restrictive diet leading to significant physical and/or psychosocial sequelae. Largely owing to the phenotypic heterogeneity, the underlying pathophysiological mechanisms are relatively unknown. Recently, the communication between microorganisms within the gastrointestinal tract and the brain—the so‐called microbiota‐gut‐brain axis—has been implicated in the pathophysiology of eating disorders. This Spotlight review sought to investigate and conceptualize the possible ways that the microbiota‐gut‐brain axis is involved in ARFID to drive future research in this area.

**Method:**

By relating core symptoms of ARFID to gut microbiota and its signaling pathways to the brain, we evaluated how the gut microbiota is potentially involved in the pathophysiology of ARFID.

**Results:**

We hypothesized that the restricted type and amount of food intake characteristic of ARFID diminishes gut microbial diversity, including beneficial bacteria and their metabolites capable of signaling to the brain, to modulate biopsychological pathways relevant to ARFID: homeostatic signaling, food reward, interoception, sensory sensitivity, disgust, perseveration, fear‐based learning, and mood. Candidate signaling mechanisms include microbial‐induced effects on inflammation, cortisol, and neurotransmitters such as dopamine and serotonin.

**Discussion:**

Through reviewing the extant evidence, we conceptualized a new theoretical framework of ARFID with an emphasis on microbiota‐gut‐brain axis signaling to inform future research. Although more research is necessary to evaluate this theoretical model, the tentative evidence suggests that therapeutics specifically targeting the gut microbiota for the treatment of ARFID symptomatology warrants more investigation.

**Summary**

- The bidirectional communication between the microorganisms inhabiting the gastrointestinal tract and the brain along the microbiota‐gut‐brain axis is known to influence feeding and eating behavior.
- However, the role of this axis in the development and maintenance of avoidant/restrictive food intake disorder (ARFID), an eating disorder characterized by restrictive eating, has yet to be investigated.
- Consideration of this potential mechanistic pathway has promise to foster new research and inform novel treatments for ARFID.

## 1 Introduction

Avoidant/Restrictive Food Intake Disorder (ARFID) is characterized by a persistent failure to meet adequate nutritional and/or energy needs due to restricted food intake, which results in severe physical and/or psychosocial consequences (American Psychiatric Association, 2013). Dietary restriction in ARFID manifests as limited amount and/or variety of food intake and can be driven by a range of factors including complaints of feeling full, sensory‐based avoidance, neophobia, and fears of swallowing, choking, and/or vomiting (Norris et al. 2014; Sanchez‐Cerezo et al. 2024; Schmidt et al. 2018). Notably, restriction is not motivated by body image disturbances or weight‐loss intentions. Likely due to the heterogeneity in etiology, the causal mechanisms that underlie ARFID are still poorly understood. Intriguingly, there is growing support that the gut microbiota (the vast community of microorganisms within the gastrointestinal tract that communicate bidirectionally with the brain along the microbiota‐gut‐brain axis to influence feeding, emotion, and cognition) is involved in modulation of brain and behavior and has been implicated in the pathogenesis of eating disorders (Carbone et al. 2021; Guo and Xiong 2024; Rantala et al. 2019; Terry, Barnett, and Gibson 2022).

The microbiota‐gut‐brain axis comprises pathways through which the gut and brain communicate including vagal, immune, and endocrine signaling (Cryan et al. 2019). Additionally, microbial metabolites, such as short‐chain fatty acids (SCFAs; e.g., butyrate) produced by microbial fermentation of dietary fiber, also possess neuromodulatory functions to influence brain function. Although several factors modify microbiota‐gut‐brain axis signaling, diet is among the most profound manipulators (Johnson et al. 2019; Schneider, O'Riordan et al. 2024). Indeed, a healthy diet rich in various macro‐ and micronutrients nurtures a diverse gut microbiota with high functional capacity to digest and synthesize nutrients, alongside enrichment of microbial metabolites capable of signaling to the brain (Xiao et al. 2022). However, the diet of individuals with ARFID is often homogenous and nutrient‐depleted, suggesting potential detrimental effects on the microbiota‐gut‐brain axis that contribute to ARFID symptomatology. Moreover, the high prevalence of gastrointestinal symptoms and disorders of gut‐brain interaction in ARFID (Burton Murray et al. 2022; Eddy et al. 2015; Murray et al. 2021; Staller, Abber, and Burton Murray 2023; Weeks et al. 2023) further implicates the gut microbiota while opening up possibilities for new biomarkers.

**FIGURE 1:**
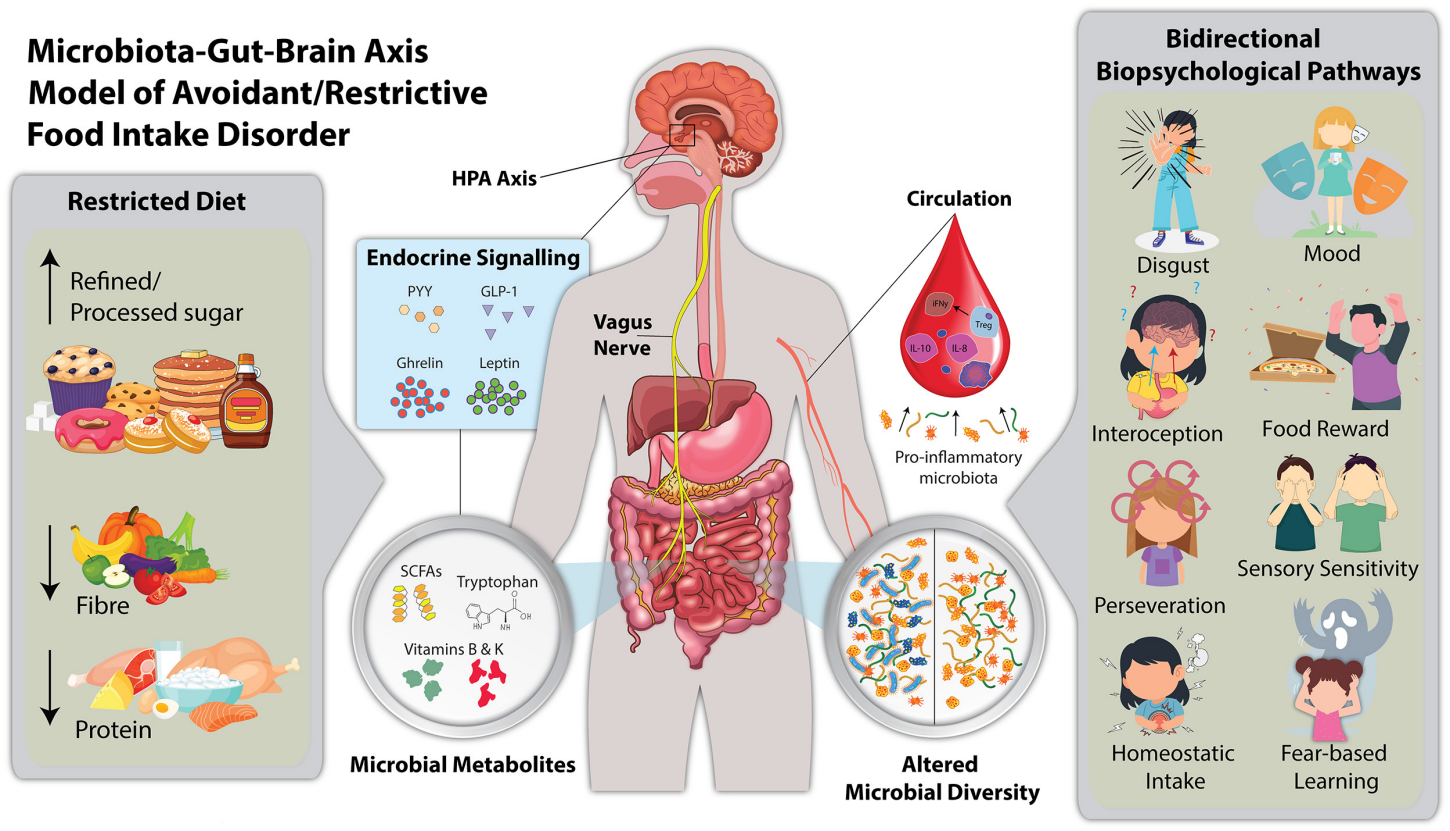
The pathophysiological microbiota‐gut‐brain‐axis model of avoidant/restrictive food intake disorder (ARFID). Evidence suggests that the microbiota‐gut‐brain‐axis plays a pathophysiological role in the onset and maintenance of symptoms of ARFID. A restricted diet in amount and variety of foods, including fiber and protein‐rich foods, promotes a specialized microbiota, thus depleting microbial diversity and their metabolites, including short‐chain fatty acids (SCFAs) and vitamins. High amounts of refined/processed sugar increase peripheral inflammation. Bidirectional biopsychological pathways through which these nutrition‐induced effects on the gut microbiota contribute to ARFID phenotypes include: homeostatic intake, food reward, interoception, sensory sensitivity, disgust, perseveration, fear‐based learning, and mood. Candidate biological mechanisms include vagal, inflammatory, and endocrinological signaling via microbial metabolites. GLP‐1: glucagon‐like peptide 1. HPA: hypothalamic–pituitary–adrenal. PYY: peptide YY.

To date, only one study has explored the relationship between gut microbiota and ARFID (Ye et al. 2023). In this observational study, 102 children aged 3–12 years with ARFID and 33 healthy control children were compared on measures of gut microbial composition and function. Bacterial diversity differed between groups and distinct microbial clustering was observed. Children with ARFID had enrichment of *Enterobacteriaceae*, *Bacteroidaceae*, *Enterobacterales*, *Bacteroides*, and *Bacteroides vulgatus* compared to controls. At the functional level, GT26, a group of glycosyltransferases involved in transferring sugar molecules, was elevated in children with ARFID. Although diet history was not reported, this finding may reflect higher carbohydrate intake that has been previously reported in children with ARFID (Harshman et al. 2019; Schmidt et al. 2021). This study provides tentative, preliminary evidence of a relationship between gut microbiota and ARFID, but more understanding of the underlying mechanisms is needed. In this **Spotlight review**, we sought to address this gap of knowledge by proposing a new theoretical model for conceptualizing the pathogenesis and maintenance of ARFID within the context of the microbiota‐gut‐brain axis (see Figure 1). This model is based on indirect evidence from neighboured fields due to the limited direct evidence for ARFID. First, we explored how dietary habits characteristic of ARFID could alter gut microbiota composition and function. By linking ARFID phenotypes to available microbiota‐gut‐brain axis findings in preclinical and clinical samples, we further suggested potential biopsychological pathways through which these diet‐induced effects on the gut microbiota might underpin ARFID symptoms. The goal of this theoretical model is to form a foundation for future research with the overall aim of deriving alternative therapeutics.

## 2 ARFID Dietary Composition and the Gut Microbiota

Individuals with ARFID have reduced fruit, vegetable, and protein intake but higher intake of added sugars and total carbohydrates (Harshman et al. 2019). This pattern of dietary intake, combined with the homogeneous diet typical of ARFID, is generally linked to poor gut microbiota health. It is well‐known that a varied diet rich in fiber, polyphenols, fermented foods, and unsaturated fatty acids consistently supports a diverse gut microbiota and the enrichment of beneficial bacteria capable of producing metabolites that signal to the brain in humans (Singh et al. 2017). Specifically, SCFAs produced from microbial metabolism of fiber are essential regulators of brain function with deficiencies linked to anxiety, depression, and anorexia nervosa (Monteleone et al. 2021; Prochazkova et al. 2021; Simpson et al. 2021). Adequate intake of protein‐rich foods is further necessary to supply the gut microbiota with a precursor to serotonin (tryptophan), a key neurotransmitter that influences feeding and emotion (Zhu et al. 2024). On the other hand, a diet that is fiber‐deficient, but high in processed and refined sugars and saturated fats, generally reduces the abundance of beneficial bacteria, stimulating peripheral and central inflammation in rodents while creating susceptibility to infection (Berding et al. 2021). Furthermore, individuals with ARFID report lower intake than controls of micronutrients, including B and K vitamins, zinc, iron, and potassium (Harshman et al. 2019; Schmidt et al. 2021). Importantly, certain gut microbes synthesize vitamins B and K, with several butyrate‐producing bacteria being dependent upon endogenous and host‐supplied B‐vitamins (Pham et al. 2021). This loss of SCFAs can have further negative consequences, given their role in mineral bioavailability through promoting iron absorption (Yilmaz and Li 2018). It has also been shown that microbial vitamin synthesis varies with malnutritional status (Gehrig et al. 2019), indicating bidirectional effects of host‐microbe nutrient supply. Such effects likely perpetuate a vicious cycle of micronutrient deficiency with implications on appetite, mood, and cognition (Benton 2008; Ghrayeb et al. 2020). Finally, iron supplementation in children has been shown to reduce the abundance of beneficial bacteria and promote peripheral inflammation (Tang et al. 2017; Yilmaz and Li 2018; Zimmermann et al. 2010), suggesting a counterproductive worsening of gut microbiota health. Given the high rates of micronutrient supplementation in ARFID, evidence‐based strategies—for example, possibly through concurrent prebiotic supplementation—for mitigating the negative effects of supplementation on gut microbiota health are needed.

## 3 Biopsychological Pathways Related to ARFID Phenotypes and Microbiota‐Gut‐Brain Axis

Some individuals with ARFID restrict intake due to feeling full, potentially reflecting disturbances in homeostatic intake signaling. Indeed, lower fasting leptin levels and increased cholecystokinin, alongside blunted postprandial ghrelin and peptide YY responses, have been shown in individuals with ARFID (Aulinas et al. 2020; Becker et al. 2021; Murray et al. 2022). Notably, SCFAs modulate leptin, ghrelin, glucagon‐like peptide‐1, and PYY secretion, while L‐tryptophan stimulates cholecystokinin, glucagon‐like peptide‐1, and peptide YY (Boscaini et al. 2022). Tryptophan has been further implicated in selection of carbohydrates, such that bacterial genes involved in the metabolism of tryptophan and plasma tryptophan availability influenced voluntary carbohydrate intake in mice (Trevelline and Kohl 2022). At the hedonic level, individuals with ARFID report apathy to eating, and increasing enjoyment to food is an objective in some cognitive‐behavioral treatments (Burton‐Murray et al. 2024; Thomas et al. 2017). Intriguingly, fecal microbiota transplantation of stool samples from obese mice into naïve mice altered hedonic feeding in recipient mice and striatal dopaminergic activity (de Wouters d'Oplinter et al. 2021). In humans, fecal microbiota‐derived indole, a tryptophan metabolite, correlated with brain activity in reward‐related areas, as well as self‐reported food addiction and anxiety (Osadchiy et al. 2018). Taken together, it is possible that reduced tryptophan availability to gut microbes as a result of inadequate protein intake could have downstream effects on food choice/intake relevant to ARFID. Disturbances in gut‐brain signaling could further implicate interoceptive unawareness and inaccuracy as a potential aetiological driver of perceived fullness in ARFID (Bonaz et al. 2021; Zucker et al. 2019). In parallel, the field can learn from previous studies linking ARFID to disorders of gut‐brain interactions, which also implicate interoceptive processes (Burton‐Murray et al. 2022; Murray et al. 2021; Weeks et al. 2023).

The sensory components of the food item also determine food intake in ARFID (Brigham et al. 2018). Sensory sensitivities to food, in addition to food neophobia and disgust, are common features of autism spectrum disorder (ASD), a disorder often comorbid with ARFID (Farag et al. 2022; Harris et al. 2019; Jayashankar and Aziz‐Zadeh 2023). There is a larger body of research linking gut microbiota composition/functionality to ASD that might provide some insight into ARFID (Gonçalves et al. 2024), including the transferral of perseverative behaviors in recipient mice transplanted with fecal microbiota from ASD donors (Sharon et al. 2019). Remarkably, it was recently shown that a restrictive diet in variety typical of ASD reduced microbial diversity and loosened stool consistency in children, thus mediating the relationship between gut microbiota and ASD (Hung and Margolis 2024; Yap et al. 2021). It will be interesting to explore if homogenous diets explain, at least partially, the comorbidity between ARFID and ASD.

Fear‐based learning plays a key role in the ARFID subtype associated with avoidance of certain foods due to previous aversive consequences of food intake, such as choking, vomiting, or allergic reactions (Reilly et al. 2019). Manipulation of the gut microbiota through transient antibiotic depletion or longer‐term absence of microbes from birth (i.e., germ‐free) induced deficits in fear extinction learning in adult mice, with restoration from intervention being dependent upon intervention in the neonatal period (Chu et al. 2019; Cowan et al. 2018; Hoban et al. 2018). In humans, a combination of SCFAs delivered to the colon protected against psychological stress‐induced cortisol increase, while butyrate alone modulated fear conditioning and extinction in adult men (Dalile et al. 2020, 2024). More research is needed to understand how aversive experiences and SCFAs interact with developmental periods in ARFID.

Prior to the inclusion of ARFID as a standalone eating disorder in 2013, a subtype of childhood eating disorders was described as “food avoidance emotional disorder” wherein anxiety and depression occurred with restricted feeding (Nicholls and Bryant‐Waugh 2009). Such comorbidities continue to be observed following introduction of the current guidelines for ARFID diagnosis (Brosig et al. 2023), indicating another possible ARFID phenotype with connections to gut microbiota. Indeed, there is consistent data demonstrating robust alterations in mood in germ‐free rodents (Cryan et al. 2019), and depressive symptoms have been successfully attenuated in two patients with treatment‐resistant depression following fecal microbiota transplantation from a euthymic donor (Doll et al. 2022).

## 4 Moderating Risk Factors

Biopsychological risk factors increasing the likelihood of ARFID are likely to moderate the relationship between gut microbiota and ARFID. Fundamentally, there is a genetic component to both the expression of ARFID (Dinkler et al. 2023) and the gut‐brain crosstalk (Cheng et al. 2023), which is likely to play a critical role in this relationship. Age of onset could be another key factor. Colonization of the gut microbiota begins at birth and is highly malleable in childhood until gradually reaching stable composition into adulthood (Cryan et al. 2019). Coinciding with this colonization, food neophobia frequently peaks from ages 2 to 6 (Dovey et al. 2008). Given the robust link between food diversity and microbial diversity, restricted food *variety* in the early years is proposed to have a larger effect on gut microbiota than restricted food *amount*, although quantity has also been shown to alter microbial composition (Zmora, Suez, and Elinav 2019). It is thus possible that early life neophobia and homogenous diet limits nutrient delivery to the developing gut microbiota, consequently permanently shaping composition/functionality. Evaluation of this hypothesis may explain why individuals with longer‐term symptoms have poorer prognosis than counterparts with shorter‐term symptoms (Zimmerman and Fisher 2017). In keeping with this hypothesis, children with ARFID who consumed a greater variety of foods during childhood would be expected to have a more diverse gut microbiota and therefore less severity of symptoms. The addition of psychosocial stressors during these critical periods of development could exacerbate symptom development. For example, early‐life stress has been shown to alter gut microbiota, corticosterone, and systemic immune responses in mice (O'Mahony et al. 2009). Adverse experiences, including childhood abuse, trauma, invasive postnatal procedures (e.g., gastric tube), or parental pressure to eat in response to avoidant/restrictive food intake and low bodyweight (Brosig et al. 2023; Eichler et al. 2019; Mitchell et al. 2024), are therefore predicted to increase symptom severity.

## 5 Discussion

As in many disorders that have implicated the microbiome, a key question in evaluating the role of the gut microbiota in ARFID is cause or consequence. Given the individual nature of the gut microbiota to metabolize specific foods (Johnson et al. 2019), it is possible that baseline microbial composition predisposes individuals to ARFID. Longitudinal designs that track individuals prior to symptom onset will be critical in determining directionality. A recent systematic review reported a prevalence range of ARFID symptoms in general populations ranging from 0.3% to 15.5% (Sanchez‐Cerezo et al. 2023). Prospective, population‐based cohorts that follow individuals from birth with longitudinal stool samples and eating behavior measures and/or food intake data (e.g., LIFE Child Study (Germany; *N* = 3000) (Poulain et al. 2017), Generation R Study (Netherlands; *N* = 9000), Danish National Birth Cohort (Denmark; *N* = 100,000)) are positioned to capture this prevalence. The data from these cohorts should be leveraged to determine the causal role of the gut microbiota in avoidant/restrictive food intake relevant to ARFID. Randomized controlled trials with nutrition (e.g., fiber, protein) or supplementation intervention can further add valuable insights about causality of the gut microbiota. Large sample sizes are needed to link phenotypes with microbial composition and add clarity to the heterogeneity characteristic of ARFID. Multimodal outcomes including self‐report mood, nutritional intake, eating behavior, and brain imaging (e.g., positron emission tomography imaging for dopamine signaling) are necessary to provide a comprehensive overview of pathophysiology. Biological sampling of gut microbiota composition and their metabolites, inflammation, and cortisol is essential for determining mechanisms of action. Finally, preclinical models can also be exploited to gain both causal and mechanistic insights not otherwise possible in humans. For example, assessment of phenotype transferal from fecal microbiota transplantation of human donors with ARFID into naïve recipient rodents would untangle directionality and inform causality.

### 5.1 Implications for Novel Therapeutics

At present, there are no evidence‐based recommendations for ARFID treatment (American Psychiatric Association 2013). Future research that evaluates this proposed pathophysiological model of microbiota‐gut‐brain axis and ARFID could open new avenues for novel treatments that target the gut microbiota. Whole foods items that are fiber‐rich (e.g., fruits, vegetables) or fermented (e.g., yogurt, kimchi) have been shown to exert beneficial effects on the microbiota‐gut‐brain axis in clinical and nonclinical populations (Schneider, Balasubramanian et al. 2024). Moreover, whole foods modulate the gut microbiota more than individual nutrients (Johnson et al. 2019), and the effects of whole food interventions on microbial composition can be observed within 24 h (David et al. 2014). Due to the inherent difficulties with incorporating new foods in ARFID, exposure interventions to new gut‐targeted foods within clinical settings may facilitate acceptance. For treatment‐resistant individuals, pre−/probiotic supplementation may be viable alternatives. Microbial composition and functionality of stool samples pre−/post‐nutrition intervention are ideally measured via shotgun metagenomic sequencing, but practical measurements of gut transit time and stool consistency can be implemented as noninvasive and feasible measures of gut health (Asnicar et al. 2021; Vandeputte et al. 2016). For example, patients can track changes in stool consistency via the Bristol Stool Chart (Lewis and Heaton 1997) and by consuming new foods with blue dyes to determine gut transit time. This could support quick and clinically meaningful classification of newly introduced foods for the individual, parent, and/or treatment team.

Other possible treatments that target the microbiota could include stimulation of the vagus nerve—a signaling pathway between the gut and brain—which has demonstrated improvements in gastrointestinal symptoms, mood, and obesity (Goggins, Mitani, and Tanaka 2022). Exercise has also been shown to alter gastric motility (Horner et al. 2015) and improve digestive symptoms in psychiatric inpatients (Kim et al. 2014). We hypothesize that improvements in gastric health will improve symptoms in individuals with ARFID.

Taken together, viewing ARFID through the lens of the microbiota‐gut‐brain axis offers a novel approach to understanding its pathophysiology and the potential for designing new therapeutic strategies. Because of its malleable nature, changing the microbiome also offers hope to patients with this poorly understood disorder.

## Author Contributions

**Elizabeth Schneider:** conceptualization, methodology, writing – original draft, writing – review and editing. **Ricarda Schmidt:** conceptualization, methodology, writing – review and editing. **John F. Cryan:** conceptualization, methodology, supervision, writing – review and editing. **Anja Hilbert:** conceptualization, methodology, writing – review and editing.

## Conflicts of Interest

Author ES has received honorarium from Janssen Sciences Ireland UC. JFC has received research funding from Dupont/IFF Kerry Foods and has been an invited speaker at meetings organized by Bromotech. Author AH reports receiving research grants from the German Federal Ministry of Education and Research, German Research Foundation, Innovation Fund, and Roland Ernst Foundation for Health Care; royalties for books on the treatment of eating disorders and obesity with Hogrefe and Kohlhammer; honoraria for workshops and lectures on eating disorders and obesity and their treatment; honoraria as editor of the *International Journal of Eating Disorders* and the journal *Psychotherapeut*; honoraria as a reviewer from Oxford University Press and the German Society for Nutrition; and honoraria as a consultant for WeightWatchers, Zweites Deutsches Fernsehen, Takeda, and Lilly.

## Data Availability

Data sharing is not applicable to this article as no new data were created or analyzed in this study.
